# PROMOTING AGING IN PLACE VIA RESEARCH, POLICY, AND PRACTICE IN AGE-FRIENDLY COMMUNITIES

**DOI:** 10.1093/geroni/igz038.098

**Published:** 2019-11-08

**Authors:** Kathy Black

**Affiliations:** University of South Florida, Sarasota -Manatee, Sarasota, Florida, United States

## Abstract

The majority of Americans overwhelmingly prefer to age in place and in the communities in which they reside. Age-friendly communities support aging in place by focusing attention on features both inside and outside of the home. The global age-friendly community model provides a framework that requires assessing community-based older adults’ needs and preferences about, and developing subsequent action towards, features of the social, service and built environment including housing and transportation which are considered essential to aging successfully at home. This presentation discusses the intersect between research, policy and practice in an age-friendly community which utilized micro-level findings from older adults (n = 1, 172) to enact macro-level collaborations across local and statewide government and professional groups to facilitate aging in place across the domains of housing and transportation.

